# Spinal cord motor neuron plasticity accompanies second‐degree burn injury and chronic pain

**DOI:** 10.14814/phy2.14288

**Published:** 2019-12-19

**Authors:** Siraj Patwa, Curtis A. Benson, Lauren Dyer, Kai‐Lan Olson, Lakshmi Bangalore, Myriam Hill, Stephen G. Waxman, Andrew M. Tan

**Affiliations:** ^1^ Department of Neurology and Center for Neuroscience and Regeneration Research Yale University School of Medicine New Haven Connecticut; ^2^ Rehabilitation Research Center Veterans Affairs Connecticut Healthcare System West Haven Connecticut

**Keywords:** burn injury, central sensitization, dendritic spines, motor neuron, pain, reflex control

## Abstract

Burn injuries and associated complications present a major public health challenge. Many burn patients develop clinically intractable complications, including pain and other sensory disorders. Recent evidence has shown that dendritic spine neuropathology in spinal cord sensory and motor neurons accompanies central nervous system (CNS) or peripheral nervous system (PNS) trauma and disease. However, no research has investigated similar dendritic spine neuropathologies following a cutaneous thermal burn injury. In this retrospective investigation, we analyzed dendritic spine morphology and localization in alpha‐motor neurons innervating a burn‐injured area of the body (hind paw). To identify a molecular regulator of these dendritic spine changes, we further profiled motor neuron dendritic spines in adult mice treated with romidepsin, a clinically approved Pak1‐inhibitor, or vehicle control at two postburn time points: Day 6 immediately after treatment, or Day 10 following drug withdrawal. In control treated mice, we observed an overall increase in dendritic spine density, including structurally mature spines with mushroom‐shaped morphology. Pak1‐inhibitor treatment reduced injury‐induced changes to similar levels observed in animals without burn injury. The effectiveness of the Pak1‐inhibitor was durable, since normalized dendritic spine profiles remained as long as 4 days despite drug withdrawal. This study is the first report of evidence demonstrating that a second‐degree burn injury significantly affects motor neuron structure within the spinal cord. Furthermore, our results support the opportunity to study dendritic spine dysgenesis as a novel avenue to clarify the complexities of neurological disease following traumatic injury.

## Graphical abstract

Globally, more than 11 million people a year suffer burn injuries severe enough to require medical attention. Here, we present a retrospective study that a second‐degree burn injury can induce potentially adverse motor neuron synaptic plasticity within the spinal cord. When these results are interpreted in combination with our previous functional study, we conclude that even superficial cutaneous burn injury can create long‐term changes within the spinal cord parenchyma.

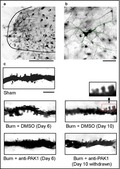

## 1 INTRODUCTION

Burn injuries and associated complications present a major public health challenge. Globally, more than 11 million people a year suffer burn injuries severe enough to require medical treatment (Latarjet & Choinere, 1995; Peck, 2011, 2012). Of these patients, many develop clinically intractable hyperexcitability disorders, for example, pain, long after healing of the initial injury is complete (Fischer and Waxman 2012). The failure to address burn injury‐associated complications is due in part to the lack of mechanistic insight into the development of changes within the central nervous system (CNS) (Guo et al., 2018).

Burn injury has been shown to contribute to several types of peripheral neuropathies associated with sensory dysfunction (Strong, Agarwal, Cederna, & Levi, 2017). Excessive peripheral nerve firing activity within scar tissue, peripheral nervous system (PNS) and CNS inflammation, loss of inhibitory regulation, and structural plasticity can contribute to chronic secondary complications associated with burn injury (Bijlard et al., 2017; Chang & Waxman, 2010; Guo et al., 2018; Shields et al., 2012). Less explored is the role of dendritic spine plasticity, specifically following burn trauma. Dendritic spines are microscopic, specialized synaptic structures that serve as sites of excitatory input and are crucial elements in normal synaptic transduction (Chen, Rex, Casale, Gall, & Lynch, 2007; Yuste & Bonhoeffer, 2001). Importantly, malformed dendritic spines in cortical tissue have been observed in a spectrum of neuropsychiatric and cognitive diseases, strongly suggesting that abnormal spine structure is directly related to abnormal function (Tan, 2015). In this retrospective study, we present data showing significant changes in dendritic spines on spinal cord motor neurons following a second‐degree burn injury.

In our previous work, we have identified a common structural motif of abnormal dendritic spine morphology strongly associated with hyperexcitability disorders, for example, pain and spasticity, following several traumatic injury events, including partial‐thickness burn injury (Bandaru, Liu, Waxman, & Tan, 2015; Guo et al., 2018; Tan et al., 2013; Wang et al., 2016; Zhao et al., 2016). However, despite circumstantial evidence demonstrating significant adverse effects in the spinal motor system after burn injury, no study has yet reported the presence of dendritic spine dysgenesis in these ventral horn motor neurons. Thermal burn or electrical burn injuries can lead to structural plasticity in the ventral horn and contribute to either the progressive loss or gain of motor tone, which may persist in some cases with amyotrophic lateral sclerosis (ALS)‐like symptoms (Higashimori, Whetzel, Mahmood, & Carlsen, 2005; Sirdofsky, Hawley, & Manz, 1991). Interestingly, in a mouse model of ALS, increased dendritic spine density in motor neurons was indicative of improved cell survival, suggesting that dendritic spine remodeling has been reported to contribute to a neuroprotective response to disease (Fogarty, Mu, Noakes, Lavidis, & Bellingham, 2016). Given our prior findings demonstrating changes in dendritic spines in the nociceptive system following burn injury (Guo et al., 2018), we performed this follow‐up investigation of spine plasticity in the motor system to clarify the cellular reorganization underlying intractable neuropathologies associated with thermal burn injury.

This postmortem investigation was made possible by leveraging the archival durability and reliability of the Golgi‐staining method. We performed a retrospective analysis of dendritic spine profiles in alpha‐motor neurons from adult mice with burn injury‐induced neuropathic pain. Mice with burn injury had a significant increase in dendritic spine density; in particular, mature mushroom‐shaped spines that have been associated with hyperexcitability in dorsal or ventral horn circuits (Bandaru et al., 2015; Tan, Choi, Waxman, & Hains, 2009). These postburn changes in dendritic spine morphology appeared to be Pak1‐signaling‐dependent, since treatment with romidepsin (an FDA‐approved Pak1‐inhibitor) restored dendritic spine profiles in motor neurons to levels similar to uninjured Sham animals. Despite the relatively short half‐life of romidepsin (~4–10 hr) (VanderMolen, McCulloch, Pearce, & Oberlies, 2011), treatment with the drug was durable. After romidepsin withdrawal, the normalized morphology of spine profiles remained constant for up to 3 days. Taken together, this study demonstrates that dendritic spine dysgenesis occurs in ventral horn motor neurons and responds to a systemically administered clinically available Pak1‐inhibitor. Future studies would help determine whether dendritic spines are a structural correlate of neurological disease, and if dendritic spine profiling could be a prognostic tool for therapeutic response.

## 2 MATERIALS AND METHODS

### 2.1 Animals

Experiments were performed in accordance with the National Institutes of Health *Guidelines for the Care and Use of Laboratory Animals* and were approved by the VA Institutional Animal Use Committee. Animals were housed under a 12 hr light/dark cycle in a pathogen‐free area with food and water provided ad libitum. Weight‐matched, adult mice were used for this retrospective study. Spinal cord tissue samples were collected from a total of 28 adult mice, which had undergone a burn injury (male/female equal mix; C57Bl6; 25 ± 1.8 g; Harlan) (Guo et al., 2018).

### 2.2 Burn Injury

For the second‐degree burn‐injury model, we used modified procedures described previously (Shields et al., 2012; Tan et al., 2013). The burn apparatus was custom designed in Fusion 360 (Autodesk) and 3D‐printed on an Ultimaker 2 + printer (Ultimaker, EU) using heat‐resistant ABS filament (see our previous study (Guo et al., 2018) for details of this apparatus). Briefly, with this burn device, we pumped hot water (75°C) continuously through an inflow opening to heat a copper plate (1‐mm thick). After traveling through this system, the water exits through outflow pipe. To ensure consistent heat application between burn‐injured animals, we monitored the temperature of the metal plate with a surface thermometer. In our prior study, before any burn‐injury (Guo et al., 2018), we first established baseline pain threshold in two assays for heat hyperalgesia and mechanical allodynia. In baseline heat hyperalgesia testing (i.e., Hargreaves method) (Dirig, Salami, Rathbun, Ozaki, & Yaksh, 1997), we observed a mean hind paw withdrawal threshold latencies between 14.5 and 15.2 s. In baseline testing for mechanical allodynia, we used systematic application of graded Von Frey filaments (Chaplan, Bach, Pogrel, Chung, & Yaksh, 1994; Guo et al., 2018). In baseline hind paw mechanical withdrawal thresholds testing, we observed a mean threshold of between 0.32 and 0.35 g (Guo et al., 2018). Following these behavioral assays for baseline pain threshold (Guo et al., 2018), we anesthetized animals with isoflurane (3%–2% vaporized in oxygen). To perform the burn injury, we pressed the glabrous surface of the left hind paw to the 75°C heated metal surface of the apparatus for 15 s with a constant 10‐g force (i.e., applied with a small sandbag on the dorsal aspect of the paw). To prevent infection, silver sulfadiazine ointment was applied to the injured site. Sham animals underwent the same procedures, but the metal surface temperature was maintained at room temperature (24 ± 1°C).

### 2.3 Romidepsin (anti‐Pak1) administration

Anti‐Pak1 or control treatment consisted of DMSO (1% in 0.01M PBS) or romidepsin (Abcam, ab143287, 5 mg/kg in 1% DMSO/0.01M PBS), respectively, that was injected i.p. over 3 days at 9:00 am everyday successively on days 4, 5, and 6 postburn and before any behavioral testing. The dosage of romidepsin was initially calculated using the FDA guidelines for converting clinical drug dosages between human and animal (animal mg/kg dose × animals surface area (km) = human mg/kg dose × human surface area (km)) from the maximum‐tolerated dose (MTD) for romidepsin for human cancer treatment (https://www.fda.gov/media/72309/download; accessed 08‐10‐18). The MTD for romidepsin use in our animals was 5 mg/kg injected i.p. twice daily. These procedures resulted in the production of five comparator groups (total animals in this study = 28): Sham + DMSO (*n* = 9); Burn + anti‐Pak1 (Day 6) (*n* = 4); Burn + DMSO (Day 6) (*n* = 4); Burn + DMSO (Day 10) (*n* = 4); Burn + anti‐Pak1 (Day 10) (*n* = 7) (see previous Guo et al., 2018 for Study Design).

### 2.4 Golgi‐staining and histology

Tissue was stained by the Golgi‐method described previously (Guo et al., 2018). Briefly, mice were killed by decapitation without fixation. Spinal cord tissues from the lumbar enlargement (L4–L5) were rapidly dissected (<5 min), rinsed in distilled water, and processed using a commercial kit (using manufacturer's instructions; FD Neurotechnologies). Twenty days later after incubation in the Golgi kit's impregnation solutions, 180‐micron thick coronal sections were cut on a vibratome (Leica VT1200S; Leica Biosystems), and mounted on gelatinized glass slides. Mounted sections were stained, rinsed in distilled water, dehydrated, cleared, and coverslipped using Permount medium. Golgi‐stained sections were visualized with a transmitted light microscope (Nikon Eclipse 80i). Images were captured with a HQ Coolsnap camera (Roper Scientific; Tucson). All analyses of alpha‐motor neurons were performed by blinded observers using the Neurolucida software suite (version 9.0; MicroBrightfield) and a pen‐monitor tablet (Cintiq 22 HD, Wacom). We analyzed the completed three‐dimensional reconstructions of motor neurons for spine density and distribution. Each imaging session consisted of a contour map (outline of the spinal cord section with location of identified neuron; data not shown) and the motor neuron, which was traced in the X‐, Y‐, and Z‐axis. Dendritic spine types were located and marked on each reconstructed dendritic branch (thin spines, blue; and mushroom spines, red). Dendritic spine density was expressed as dendritic spine number per 10‐μm dendrite length. To determine any changes in spatial distribution of dendritic spines relative to the cell body, we used a Sholl's analysis (Tan et al., 2008). Four 50‐μm wide spherical bins were formed around each cell body and spine density within each bin was averaged within each treatment group. For statistical comparison, spine density at dendrite branch locations within each bin from the cell body was pooled within each group, and compared across treatment groups.

### 2.5 Motor neuron imaging and identification

**Figure 1:**
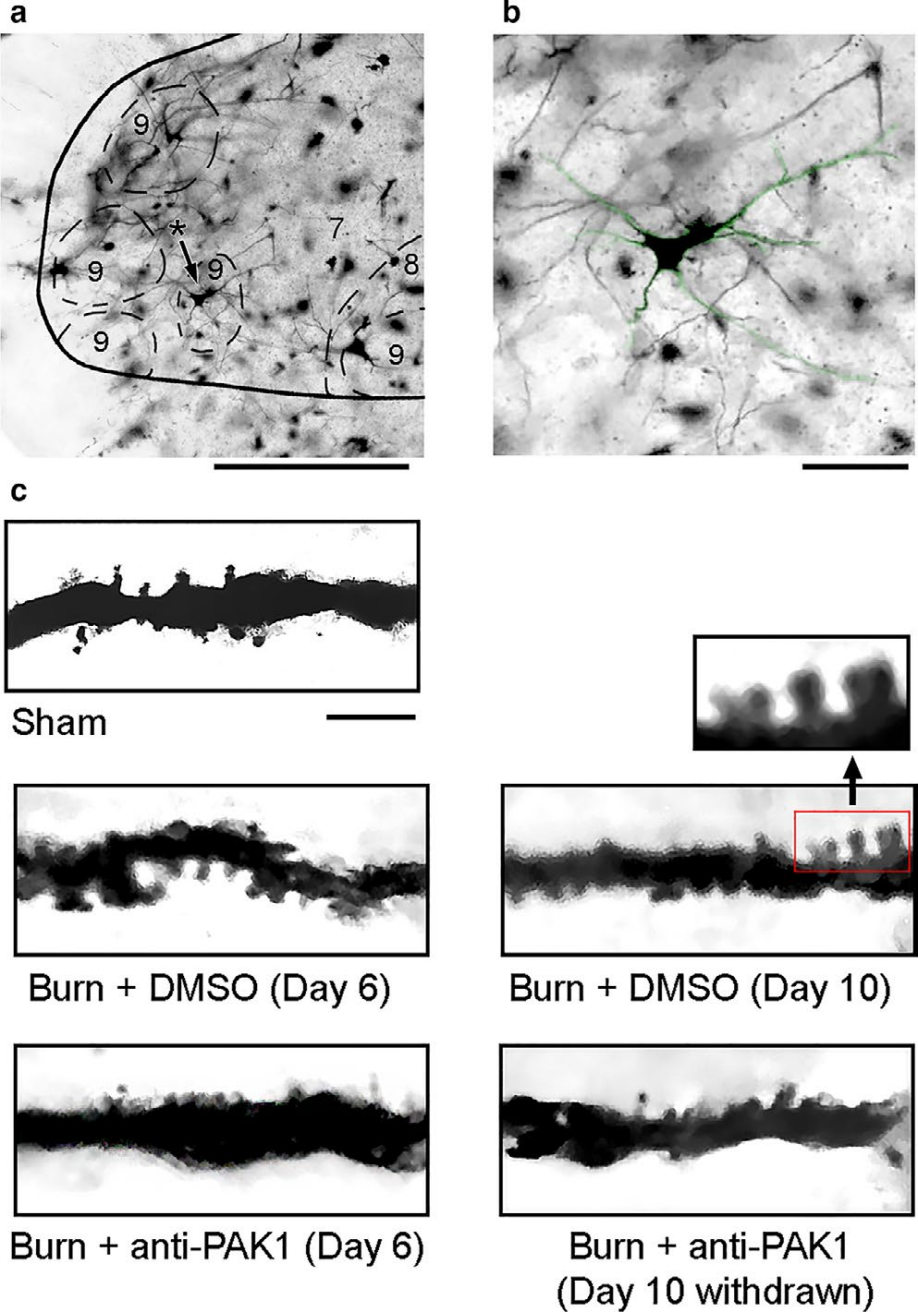
Golgi‐stained tissue from the ventral spinal cord. (a) Coronal section of spinal cord showing a representative alpha‐motor neuron within lamina IX ventral horn (asterisk with black arrow). (b) Magnified motor neuron from panel a (green highlight). (c) Dendritic branch segments from alpha‐motor neurons sampled from each group: Sham, Burn + DMSO (Day 6), Burn + DMSO (Day 10), Burn + anti‐Pak1 (Day 6), and Burn + anti‐Pak1 (Day 10 withdrawal). Magnified view of multiple dendritic spines along a sampled dendritic branch is shown from the red box in panel c. Scale bars in A = 100 μm; B = 50 μm C = 10 μm

We identified and classified alpha‐motor neuron dendritic spines using profiling information from our previous publications (Bandaru et al., 2015; Zhao et al., 2016). Investigators blinded to treatment conditions performed all imaging studies and analyses. We were specifically interested in motor neuron pools that innervated muscle groups in the hind limb, that is, plantar muscle, ipsilateral to the burn injury (spinal segmental level L4). In our previous study, to ensure that we could differentiate between the ipsilateral and contralateral side of the spinal cord, we had inserted a small metal pin longitudinally through the contralateral, ventral axis of the cord (Guo et al., 2018). In coronal tissue sections, this pin‐hole served as a histological reference. Therefore, in this retrospective study, we did not sample ventral motor neurons from the contralateral side of the spinal cord, which may have been damaged by the pin‐hole reference. To identify these α‐motor neurons, we followed a screening workflow based on data from our previous study (Tan, Chakrabarty, Kimura, & Martin, 2012) and those previously validated in rats (Crockett, Harris, & Egger, 1987; Hashizume, Kanda, & Burke, 1988; Jacob, 1998). We included approximately 1–2 motor neurons per section in our analyses using the following sampling workflow: We began with a broad sample population of neurons by identifying Golgi‐stained α‐motor neurons located in the ventral spinal cord in Rexed lamina IX and with soma diameters > 25 μm (Hashizume et al., 1988; Jacob, 1998). We narrowed our sampled α‐motor neurons to those located in ventral motor pools with similar dorsolateral coordinates of motor pools known to innervate the plantar muscle as shown by retrograde tracing (Crockett et al., 1987; Jacob, 1998). As a refinement step for analysis a priori, we only included α‐motor neurons for analysis that had (a) dendrites and dendritic spines that were clearly and completely impregnated, (b) neurons that had dendritic branches appearing as a continuous length for at least 200 μm within the tissue slice, and (c) with at least one‐half of the primary dendritic branches that remained within the thickness of the tissue section, such that their endings were not cut and appeared to taper into a complete ending (see representative neuron in Figure 1). To determine if there were any morphological differences across our sample neurons, we used NeuroExplorer software (MicroBrightfield) to measure maximum cell diameter, number of primary dendrites, and their percentage of primary dendrites with secondary branches, and compared these morphometry values across treatment groups (Table 1). To refine our identification and measurements of dendritic spines, specific morphological characteristics were used (Kim, Dai, McAtee, Vicini, & Bregman, 2006; Tan, Samad, et al., 2012). Briefly, a protrusion from the dendrite branch was considered a spine only if a clear neck structure was present. If no clear neck structure was observed, then the protrusion was considered a spine when we observed concave indentations on both sides of the juxtaposed protrusion and the dendritic branch. Classification of dendritic spines into “thin” and “mushroom” morphologies was performed as follows: spines with head‐like enlargements with diameter less than the length of the neck were considered thin spines, whereas spines with head diameters exceeding the neck length were considered mushroom spines (Zhao et al., 2016). Neck length was defined as the length from the spine‐dendrite branch junction to the base of the spine head. These criteria for two spine geometric categories were used because classification into only two spine shapes allowed us to use simple, but very strict rules in classifying spine morphology. Although this approach prevented the discrimination of subtle variations in spine shape, it allowed the collection of a very large sample size of spine shapes that we and others have described physiologically characteristics of thin and mushroom spine shapes on neuronal and circuit functions (Bourne & Harris 2007; Calabrese, Wilson, & Halpain, 2006; Holmes, 1990; Tan et al., 2009). Note that these criteria do not imply the physiological characterization of the neurons we analyzed, but rather control for morphological diversity within the sampled spinal motor neuron population (Kitzman, 2005; Tashiro & Yuste, 2003). We identified a total of 60 motor neurons for inclusion in our analysis (2–3 cells per animal; Sham + DMSO = 20; Burn + DMSO, D6 = 10; Burn + DMSO, D10 = 10; Burn + Romidepsin, D6 = 10; Burn + Romidepsin, D10 = 10).

**Table 1:** Cell body dimensions and dendritic branch morphology comparison

	Maximum diameter of cell body (µm)	# of Primary dendrites	Primary dendrites with secondary branches (%)
Sham	51.2 ± 6.7	5.2 ± 1.3	49.3 ± 19.5
Burn + DMSO (D6)	52.8 ± 10.1	4.4 ± 1.6	42.1 ± 16.4
Burn + anti‐Pak1(D6)	50.8 ± 10.0	5.8 ± 1.6	44.8 ± 17.6
Burn + DMSO (D10)	45.8 ± 7.8	4.1 ± 1.0	58.7 ± 21.2
Burn + anti‐Pak1(D10)	50.6 ± 6.4	5.2 ± 1.6	41.5 ± 15.6

Note

To determine if there were any morphological differences across our sample neurons, we used NeuroExplorer software (MicroBrightfield, Williston, VT) to measure maximum cell diameter, number of primary dendrites, and their percentage of primary dendrites with secondary branches, and compared these morphometry values across treatment groups. Data are mean ± standard deviation (*SD*).

No statistical differences in any comparisons across groups (*p* > .05).

### 2.6 Statistical analysis

All statistical tests were performed at the α‐level of significance of .05 by two‐tailed analyses using parametric or nonparametric tests, as appropriate. Specifically, if comparator datasets showed a normal distribution, we employed a Student's *t*‐test for significance. If normality was not validated, we used the Mann–Whitney rank sum test. To correct for possible repeated measure errors in comparisons across the three treatment groups, we performed either Bonferroni's or Dunn's post hoc tests. Our neuronal sample sizes were determined based on our previous studies, which demonstrated sufficient power with at least 10 neurons to confidently determine significant differences with multiple treatment variables, for example, burn injury with or without anti‐Pak1 treatment. We included *n* = 10–20 motor neurons/comparator group in all comparisons shown in Figures 3 and 4. We did not compare data across the two time points (i.e., Day 6 or Day 10 postburn injury) due to a lack of statistical power. Data management and statistical analyses were performed using SigmaPlot (version 12.5; Systat Software Inc.) and Microsoft Office Excel (2018). Numerical data in the text and graphs are presented as mean ± standard error of the mean (*SEM*). Data in Table 1 are presented as mean ± standard deviation (*SD*).

## 3 RESULTS

### 3.1 Pak1 activity contributes to increased dendritic spine density in motor neurons after burn injury

**Figure 2:**
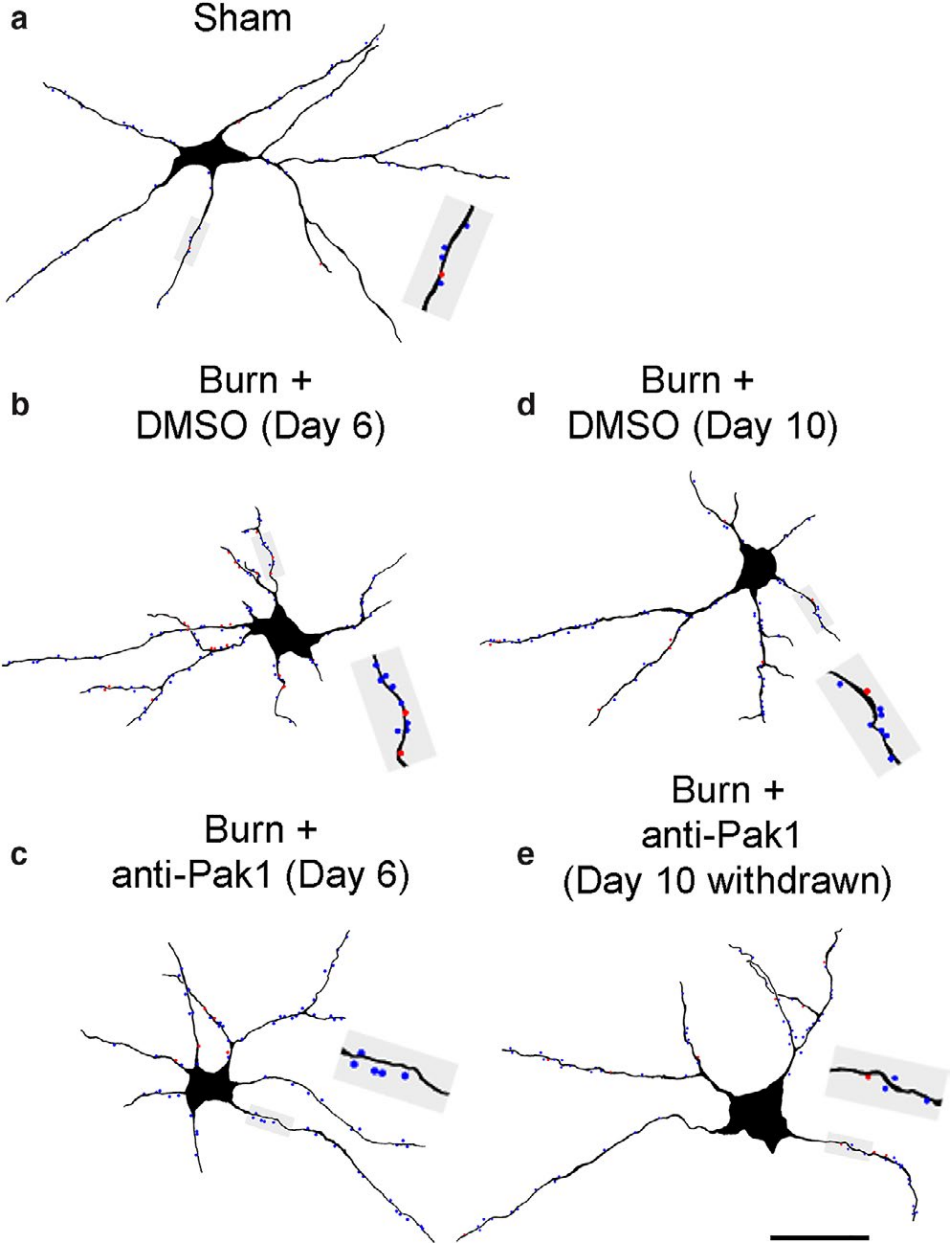
Neurolucida digital reconstruction of alpha‐motor neurons. Digital rendering shows the morphology of a representative alpha‐motor neuron from (a) Sham, (b) Burn + DMSO (Day 6), (c) Burn + anti‐Pak1 (Day 6), (d) Burn + DMSO (Day 10), and (e) Burn + anti‐Pak1 (Day 10 withdrawal). Gray shaded boxes in each panel show a magnified view of a dendritic branch segment with thin (blue dots) and mushroom (red dots) dendritic spines. Scale bars a–c = 50 μm

We identified alpha‐motor neurons from Golgi‐stained spinal cord tissue using topographical and morphological criteria (Crockett et al., 1987; Hashizume et al., 1988; Jacob, 1998; Tan, Samad, et al., 2012) (Figures 1 and 2). As shown in Figure 1a and b, we sampled motor neurons within lamina IX ipsilateral to the burn injury. These alpha‐motor neurons had cell bodies that were more than 25 microns in diameter and had extensive dendritic branches (at least 200 microns in length). We collected spinal cord tissue from animals on day 6 or 10 after burn injury. Control tissue was collected from animals 10 days following a Sham procedure (i.e., no burn injury). In Figure 1c, representative dendritic branch segments from sample neurons show dendritic spines across the five groups.

For digital reconstruction of motor neurons, we first traced the contours of the spinal cord and gray matter to document the topographical location of sampled neurons (contours not shown; representative tissue section in Figure 1a). We sampled ipsilateral alpha‐motor neurons in three‐dimensions using Neurolucida software and marked thin‐ and mushroom‐shaped dendritic spines with blue or red dots, respectively (Figure 2a–e). We did not observe significant differences in other motor neuron anatomical features across comparator groups (Table 1).

In our previous study of alpha‐motor neurons in the ventral horn, total, thin‐, and mushroom‐shaped dendritic spine densities increased following a contusion spinal cord injury (SCI) (Bandaru et al., 2015). Dendritic spines also redistributed spatially on dendritic branch locations closest to the cell body. Importantly, these dendritic spine remodeling profiles (which we term “dendritic spine dysgenesis”) in either nociceptive sensory interneurons or alpha‐motor neurons were strongly associated with increased nociceptive or spinal motor reflex excitability (Bandaru et al., 2015; Zhao et al., 2016). Thus, in this retrospective analysis of spinal cord tissue from animals with burn injury, we expected a similar change in dendritic spine morphology in spinal cord motor neurons.

**Figure 3:**
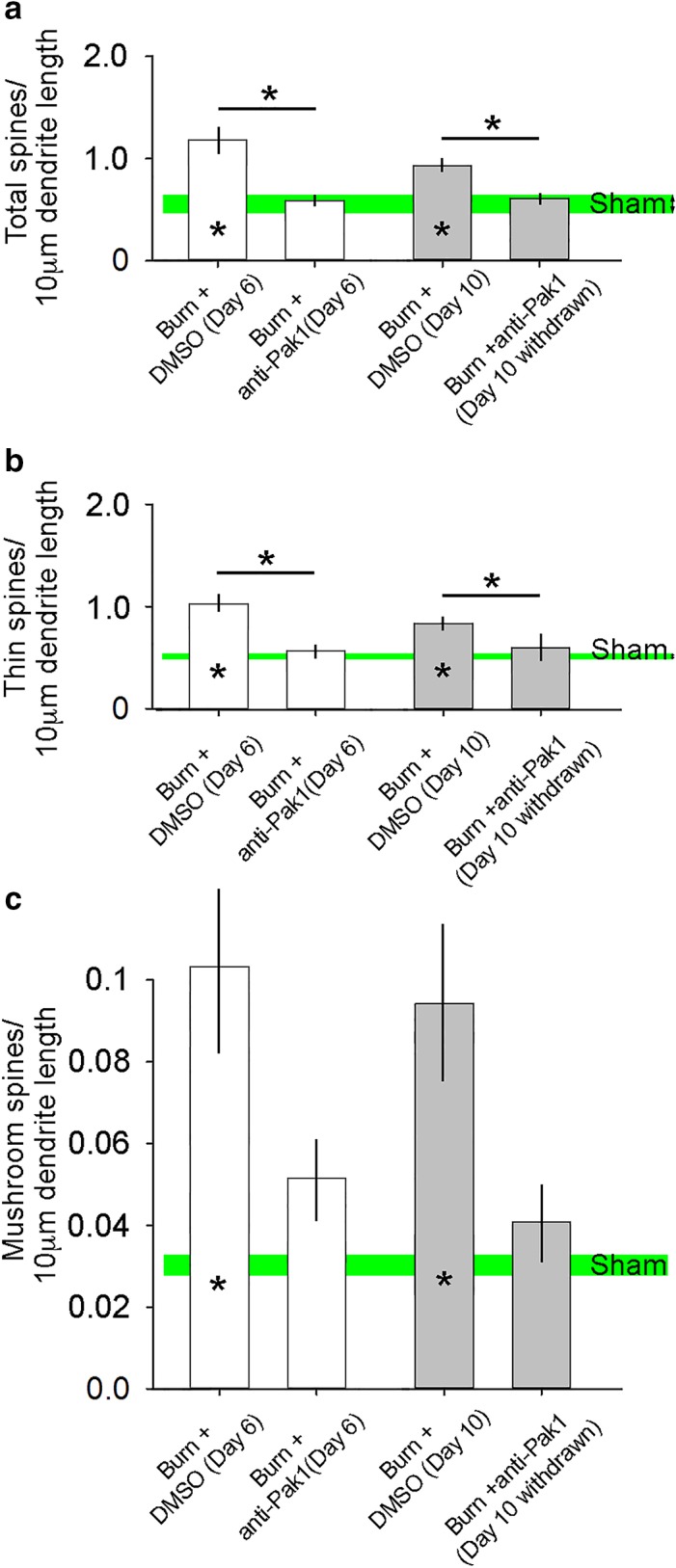
Dendritic spine density on alpha‐motor neurons. 6 days after burn injury, (a) total, (b) thin, and (c) mushroom dendritic spine density increased on ipsilateral motor neurons following second‐degree burn injury treated with control DMSO, as compared with Sham (* within bar = *p* < .05). At 10‐days postburn injury with DMSO treatment, dendritic spine density for all types also remained elevated as compared with Sham (* within bar = *p* < .05). Although treatment with anti‐Pak1, romidepsin, reduced total and thin‐shaped dendritic spine density at Day 6 and following the drug's withdrawal by Day 10 following burn injury, romidepsin had no significant effect on mushroom‐shaped dendritic spines following burn injury on alpha‐motor neurons. In general, there was no difference in dendritic spines in burn‐injured animals treated with anti‐Pak1‐inhibitor and Sham animals (n.s.). Data are shown as mean ± *SEM*

As shown in Figure 3, spine density for each treatment group was expressed as the number of all total, thin‐, or mushroom‐shaped spines per 10‐μm dendritic length, pooled within groups, and statistically compared across treatment groups. In agreement with our previous observations, we demonstrate that 6 days following a second‐degree burn injury and DMSO control treatment there was a significant increase in total (Figure 3a), thin‐ (Figure 3b) and mushroom‐shaped (Figure 3c) dendritic spine densities on ipsilateral alpha‐motor neurons, as compared with Sham (*p* < .01, Student's *t*‐test; Sham vs. Burn + DMSO (Day 6); total spines: 0.60 ± 0.13 vs. 1.1 ± 0.32 total spines/10‐μm dendrite; thin spines: 0.56 ± 0.13 vs. 1.0 ± 0.26 thin spines/10‐μm dendrite; mushroom spines: 0.03 ± 0.02 vs. 0.11 ± 0.08 mushroom spines/10‐μm dendrite). Increased total and thin‐shaped dendritic spine densities remained statistically significant in the 10‐day postburn cohort (*p* < .01, Student's *t*‐test; Sham vs. Burn + DMSO (Day 10); total spines: 0.60 ± 0.13 vs. 0.96 ± 0.17 total spines/10‐μm dendrite; thin spines: 0.56 ± 0.13 vs. 0.86 ± 0.17 thin spines/10‐μm dendrite; mushroom spines: 0.03 ± 0.02 vs. 0.09 ± 0.07 mushroom spines/10‐μm dendrite).

In experimental second‐degree burn injury, Rac1‐Pak1 pathway activity is necessary for dendritic spine remodeling within the nociceptive system and the presentation of neuropathic pain (Guo et al., 2018; Tan et al., 2013). Relevant to this report, we have previously demonstrated that romidepsin can reduce neuropathic pain symptoms following burn injury (Guo et al., 2018). Notably, Pak1 activity contributes to cytoskeletal reorganization underlying dendritic spine plasticity and has been implicated in a spectrum of neurological diseases and disorders (Allen et al., 2009; Kichina, Goc, Al‐Husein, Somanath, & Kandel, 2010; Ma, Yang, Frautschy, & Cole, 2012; Nikolic, 2008). Importantly, although we have identified Pak1 as a druggable target for managing neuropathic pain (Guo et al., 2018), no study has shown the role of Pak1 kinase in structural plasticity in the spinal motor reflex system (Gao, Dickerson, Guo, Zheng, & Zheng, 2004; Shin, Kim, Roy, & Kim, 2013; Tan et al., 2008). To assess whether inhibition of Pak1 activity would be effective in disrupting abnormal dendritic spine remodeling after cutaneous burn injury, we analyzed dendritic spines on motor neurons in adult mice treated with romidepsin, a Pak1‐inhibitor, or DMSO control at Day 6 postburn injury, and at Day 10 following drug treatment withdrawal.

In all animals with burn injury treated with romidepsin, we observed a significant reduction in burn injury‐induced dendritic spine density (all types) that was similar to Sham animals (*p *> .05; Student's *t*‐test, Sham vs. Burn + anti‐Pak1 (Day 6); total spines: 0.60 ± 0.13 vs. 0.59 ± 0.19 total spines/10‐μm dendrite; thin spines: 0.56 ± 0.13 vs. 0.53 ± 0.16 thin spines/10‐μm dendrite; mushroom spines: 0.03 ± 0.02 vs. 0.05 ± 0.05 mushroom spines/10‐μm dendrite) (Figure 3a–c). For total and thin‐shaped dendritic spines (Figure 3a and b), treatment with romidepsin significantly reduced dendritic spine densities as compared with DMSO‐treated burn‐injured animals (*p* < .05, Student's *t*‐test; Burn + DMSO (Day 6) vs. Burn + anti‐Pak1 (Day 6); total spines: 1.11 ± 0.32 vs. 0.59 ± 0.19 total spines/10‐μm dendrite; thin spines: 1.0 ± 0.26 vs. 0.53 ± 0.16 thin spines/10‐μm dendrite). There was no significant difference in mushroom‐shaped dendritic spines between romidepsin anti‐Pak1 and DMSO‐treated animals (*p *> .05; Student's *t*‐test, Burn + DMSO (Day 6) vs. Burn + anti‐Pak1 (Day 6); mushroom spines: 0.11 ± 0.08 vs. 0.05 ± 0.05 mushroom spines/10‐μm dendrite).

Interestingly, despite drug withdrawal that occurred immediately after treatment on Day 6, we observed a durable effect of romidepsin treatment, as shown at the 10‐days postburn endpoint. Specifically, we show no difference in total or thin‐shaped dendritic spine densities in comparisons between animals treated with romidepsin or DMSO (*p *> .05; Student's *t*‐test, Sham vs. Burn + anti‐Pak1 (Day 10 withdrawn); total spines: 0.60 ± 0.13 vs. 0.59 ± 0.31 total spines/10‐μm dendrite; thin spines: 0.56 ± 0.13 vs. 0.55 ± 0.29 thin spines/10‐μm dendrite). The durable effect of romidepsin treatment in reducing abnormal total and thin‐shaped spine density was also supported in comparisons with DMSO‐treated burn‐injured animals (*p* < .05; Student's *t*‐test, Burn + DMSO (Day 10) vs. Burn + anti‐Pak1 (Day 10 withdrawn); total spines: 1.0 ± 0.17 vs. 0.59 ± 0.31 total spines/10‐μm dendrite; thin spines: 0.86 ± 0.17 vs. 0.55 ± 0.29 thin spines/10‐μm dendrite). In general, romidepsin treatment had a lasting effect for mushroom‐shaped dendritic spine density when compared with Sham. Mushroom spine density was similar between animals treated with romidepsin that was withdrawn and Sham animals at 10 days postburn injury (*p *> .05; Student's *t*‐test, Sham vs. Burn + anti‐Pak1 (Day 10 withdrawn); mushroom spines: 0.03 ± 0.02 vs. 0.04 ± 0.05 mushroom spines/10‐μm dendrite). Treatment with romidepsin did not have a statistically sufficient effect on mushroom‐shaped spine density as compared with DMSO‐treated animals at Day 10 postburn injury (*p *> .05; Student's *t*‐test, Burn + DMSO (Day 10) vs. Burn + anti‐Pak1 (Day 10 withdrawn); mushroom spines: 0.1 ± 0.07 vs. 0.04 ± 0.05 mushroom spines/10‐μm dendrite). Taken together, these datasets demonstrate that burn injury results in a predictable increase in dendritic spine density (all types), which can be partially reversed with romidepsin Pak1‐inhibitor treatment. Furthermore, our results show that Pak1‐inhibitor treatment has a durable effect, since withdrawal of romidepsin treatment does not lead to a relapse in dendritic spine dysgenesis (e.g., increased density).

### 3.2 Dendritic spines exhibit most dynamic plasticity along proximal dendritic branch regions from the soma

The spatial distribution of spines along dendritic branches has a significant impact on neuronal excitability (Tan et al., 2009). Excitatory afferent inputs into dendritic branches closest to the cell body have a higher probability for generating action potentials than those inputs synapsing farther away (Holthoff & Tsay, 2002; Holthoff, Tsay, & Yuste, 2002; Rall, Burke, Smith, Nelson, & Frank, 1967; Yuste & Urban, 2004). Computer simulations have also shown that development from immature, thin‐shaped spines to mature, mushroom‐shaped spines can lead to an amplification of excitatory postsynaptic potential (EPSP) peak amplitude, for example, increased excitability (Tan et al., 2009).

**Figure 4:**
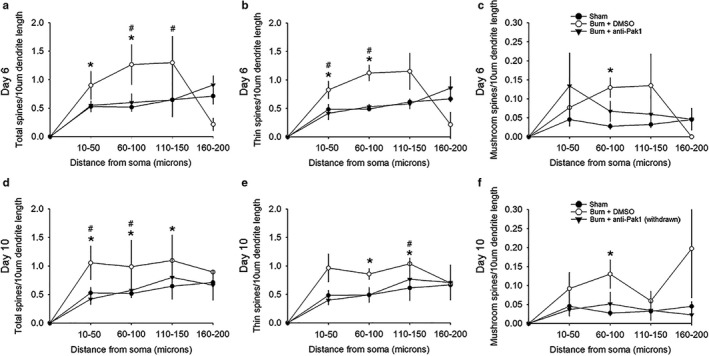
Spatial distribution of dendritic spines. (a) Total, (b) thin‐, and (c) mushroom‐shaped dendritic spines on alpha‐motor neurons increased in the two closest regions within 100 μm away from the cell body 6 days following burn injury with control DMSO treatment, as compared with Sham (*p < .05). Treatment with anti‐Pak1‐inhibitor in burn‐injured animals significantly reduced total and thin‐shaped dendritic spine densities as compared with DMSO‐treated animals (^#^ *p* < .05). As shown in panel c, there was no effect of romidepsin anti‐Pak1 treatment on mushroom‐shaped dendritic spines on alpha‐motor neurons, in comparisons with DMSO‐treated burn‐injured animals or Sham (n.s.). Following withdrawal drug and assessing dendritic spine profiles 10 days following burn injury, most dendritic spine densities remained elevated in regions closest to the cell body for (d) total, (e) thin‐, and (f) mushroom‐shaped dendritic spines as compared with Sham (*p < .05). As shown in data from Day 10 postburn injury, treatment with anti‐Pak1‐inhibitor continued to be partly effective in reducing total and thin‐shaped dendritic spines despite drug withdrawal, as compared with Sham (^#^ *p* < .05). As with Day 6, romidepsin anti‐Pak1 inhibition had no effect on mushroom‐shaped dendritic spines in the Day 10 dataset (n.s.). In animals treated with romidepsin, dendritic spine densities for all types were not significant in comparisons with Sham, suggesting that anti‐Pak1 treatment restored and maintained close‐to‐normal spine densities on alpha‐motor neurons. Data are shown as mean ± *SEM*

To investigate the spatial distribution profile of dendritic spines on motor neurons ipsilateral to the burn injury, we performed a Sholl's analysis on reconstructed sampled motor neurons. We pooled dendritic spine densities within four regions located up to 200 μm away from the cell body (see Methods and Materials) (Figure 4). On Day 6 following burn injury, our results show a significant increase in total and thin‐shaped dendritic spine densities within the 10–50 μm region in a comparison between burn‐injured animals treated with DMSO and Sham (Figure 4a–b) (*p < .05, Mann–Whitney rank sum test; Sham vs. Burn + DMSO (Day 6); 10–50 μm region; total spines: 0.53 ± 0.42 vs. 0.9 ± 0.49 total spines/10‐μm dendrite; thin spines: 0.48 ± 0.39 vs. 0.8 ± 0.48 thin spines/10‐μm dendrite). Treatment with romidepsin in burn‐injured animals reduced total dendritic spine density in the 60–100 μm and 110–150 μm regions as compared with DMSO (^#^ *p* < .05, Mann–Whitney rank sum test; Burn + DMSO vs. Burn + anti‐Pak1; 60–100 μm region; total spines: 1.3 ± 0.44 vs. 0.6 ± 0.17 total spines/10‐μm dendrite; 110–150 μm: 1.1 ± 0.37 vs. 0.64 ± 0.29 total spines/10‐μm dendrite). Similarly, for thin‐shaped dendritic spines, we observed a significant decrease with romidepsin treatment as compared with DMSO in the 10–50 μm and 60–100 μm regions (^#^ *p* < .05, one‐way ANOVA; Burn + DMSO vs. Burn + anti‐Pak1; 10–50 μm region; thin spines: 0.82 ± 0.48 vs. 0.41 ± 0.35 thin spines/10‐μm dendrite; Mann–Whitney rank sum test; 60–100 μm: 1.1 ± 0.43 vs. 0.53 ± 0.13 thin spines/10‐μm dendrite). For mushroom‐shaped dendritic spines at Day 6 postburn (Figure 4c), we observed an increase in spine densities within the 60–100 μm region in a comparison between burn‐injured animals treated with DMSO vehicle and Sham (*p < .05, one‐way ANOVA; Sham vs. Burn + DMSO; 60–100 μm region; mushroom spines: 0.03 ± 0.03 vs. 0.13 ± 0.08 mushroom spines/10‐μm dendrite). Romidepsin treatment had no effect on mushroom‐shaped spines in any regions, in comparisons with other groups (*p *> .05).

At Day 10 following burn injury (and after romidepsin drug withdrawal), we further analyzed the spatial distribution of dendritic spine density (Figure 4d–f). In DMSO‐treated burn‐injured animals, total dendritic spine densities on motor neurons remained elevated as compared with Sham in three dendrite regions, 10–50 μm, 60–100 μm, and 110–150 μm (*p < .05, Mann–Whitney rank sum test; Sham vs. Burn + DMSO; 10–50 μm region; total spines: 0.53 ± 0.42 vs. 1.1 ± 0.82 total spines/10‐μm dendrite; Student's *t*‐test; 60–100 μm region: 0.52 ± 0.16 vs. 0.99 ± 0.33 total spines/10‐μm dendrite; Mann–Whitney rank sum test; 110–150 μm region; total spines: 0.8 ± 1.0 vs. 1.1 ± 0.37 total spines/10‐μm dendrite) (Figure 4d). For thin‐shaped dendritic spine density, burn injury with DMSO treatment remained increased in comparison with Sham at two regions, 60–100 μm and 110–150 μm (*p < .05, Mann–Whitney rank sum test; Sham vs. Burn + DMSO; 60–100 μm region; thin spines: 0.49 ± 0.15 vs. 0.86 ± 0.3 thin spines/10‐μm dendrite; *t*‐test; 110–150 μm region: 0.61 ± 0.18 vs. 1.03 ± 0.32 thin spines/10‐μm dendrite) (Figure 4e). For mushroom‐shaped dendritic spines at Day 10 postburn (Figure 4f), increased spine density remained in the 60–100 μm region in a comparison between burn‐injured animals treated with DMSO and Sham (*p < .05, Mann–Whitney rank sum test; Sham vs. Burn + DMSO; 60–100 μm region; mushroom spines: 0.03 ± 0.03 vs. 0.13 ± 0.12 mushroom spines/10‐μm dendrite).

Despite romidepsin treatment withdrawal, total dendritic spine densities remained below those seen in DMSO‐treated burn‐injured animals in two regions, 10–50 μm and 60–100 μm (^#^ *p* < .05, Mann–Whitney rank sum test; Burn + DMSO vs. Burn + anti‐Pak1; 10–50 μm region; total spines: 1.1 ± 0.82 vs. 0.42 ± 0.29 total spines/10‐μm dendrite; Student's *t*‐test; 60–100 μm: 0.99 ± 0.33 vs. 0.57 ± 0.38 total spines/10‐μm dendrite) (Figure 4d). For thin‐shaped dendritic spines, romidepsin also remained effective despite treatment withdrawal in maintaining lower dendritic spine density in the 110–150 μm region (^#^ *p* < .05, Student's *t*‐test Burn + DMSO vs. Burn + anti‐Pak1; 110–150 μm: 1.0 ± 0.32 vs. 0.77 ± 0.99 thin spines/10‐μm dendrite) (Figure 4e). We observed no effect of the anti‐Pak1 drug on mushroom‐shaped spines in any regions, in comparisons with either Sham or DMSO‐treated animals on Day 10 (*p *> .05) (Figure 4f). Finally, at the Day 10 endpoint, we observed no difference in comparisons between anti‐Pak1‐treated animals and Sham for any spine type (*p *> .05; Student's *t*‐test, Sham vs. Burn + anti‐Pak1 (Day 10 withdrawn)).

### 3.3 Dendritic spine changes in the dorsal horn versus ventral horn following burn injury

The increase in dendritic spine density in ventral horn motor neurons following burn injury was also observed in sensory neurons in our previous study (Guo et al., 2018). In comparison with this retrospective study, as shown in Table 2, without anti‐Pak1‐inhibitor treatment, the second‐degree burn injury resulted in a significant increase in dendritic spine density (all types) for *both* dorsal horn sensory and ventral horn motor neurons 6 days after burn. Note that in general, motor neurons overall appear to have a lower dendritic spine density than sensory neurons (Table 2). Importantly, romidepsin treatment appeared to have a greater overall effect on motor neuron postburn dendritic spine morphologies compared with spines in sensory neurons. For example, anti‐Pak1 treatment failed to restore *close‐to‐normal* spine densities in sensory neurons, that is, spine density remained elevated compared with Sham despite anti‐Pak1 treatment (Table 2; Guo et al., 2018). In contrast, in motor neurons, the same drug treatment resulted a significant decrease in motor neuron dendritic spine densities that were statistically *indistinguishable* from uninjured Sham. Moreover, anti‐Pak1 treatment on motor neuron spine morphology was more durable than its effect on sensory neurons—At Day 10, following anti‐Pak1 drug withdrawal, although sensory neuron spine densities remained elevated compared with Sham; motor neuron spine densities continued to remain close‐to‐Sham control levels. Taken together, our retrospective data combined with our previous study demonstrate that burn injury similarly affects dendritic spine density (i.e., density increases) in both sensory and motor neurons. On the other hand, anti‐Pak1 treatment is more effective in reducing postburn injury abnormal spine density morphology on motor neurons compared with sensory neurons.

**Table 2:** Retrospective view of dendritic spine density: dorsal horn sensory neurons versus ventral horn motor neurons

Treatment arm	Dorsal horn sensory neurons^a^	Ventral horn motor neurons
Total spine density		
Burn + anti‐Pak1 (Day 6)	2.8 ± 0.4^b^, ^c^	0.59 ± 0.19^c^
Burn + DMSO (Day 6)	3.5 ± 0.6^b^	1.11 ± 0.32^b^
Burn + anti‐Pak1 (Day 10)	2.9 ± 0.6^b^	0.59 ± 0.31^c^
Burn + DMSO (Day 10)	3.1 ± 1.1^b^	1.0 ± 0.17^b^
Sham	2.4 ± 0.4	0.60 ± 0.13
Thin spine density		
Burn + anti‐Pak1 (Day 6)	2.4 ± 0.3^b^, ^c^	0.53 ± 0.16^c^
Burn + DMSO (Day 6)	2.9 ± 0.7^b^	1.0 ± 0.26^b^
Burn + anti‐Pak1 (Day 10)	2.5 ± 0.4^b^	0.55 ± 0.29^c^
Burn + DMSO (Day 10)	2.6 ± 0.9^b^	0.86 ± 0.17^b^
Sham	2.1 ± 0.4	0.56 ± 0.13
Mushroom spine density		
Burn + anti‐Pak1 (Day 6)	0.43 ± 0.2^b^, ^c^	0.05 ± 0.05
Burn + DMSO (Day 6)	0.58 ± 0.2^b^	0.11 ± 0.08^b^
Burn + anti‐Pak1 (Day 10)	0.47 ± 0.2^b^, ^c^	0.04 ± 0.05
Burn + DMSO (Day 10)	0.48 ± 0.3^b^	0.1 ± 0.07^b^
Sham	2.8 ± 0.4	0.03 ± 0.02

Note

Summary table showing the retrospective datasets of burn injury on ventral horn motor neuron dendritic spine density as compared with published data on sensory neurons following second‐degree burn injury (Guo et al., 2018). Dendritic spine density is measured as number of spines per 10 mm dendritic branch length. In all comparisons, dorsal horn exhibited a greater density of dendritic spines than compared with ventral horn neurons (*p < .01).

a Datasets from previous publication Guo et al., 2018

b Denotes *statistically significant* increase in dendritic spine density following burn injury as compared with Sham control (no burn) data.

c Denotes a *statistically significant* effect of anti‐Pak1 (romidepsin) drug treatment as compared with DMSO treatment, in burn‐injured animals *within* the same time point (i.e., Day 6 or Day 10 post drug withdrawal).

## 4 DISCUSSION

Severe burn injury is a major public health crisis. Eleven million individuals a year suffer burns that require hospitalization, and many experience secondary chronic complications with intractable neuropathic symptoms. Here, we profiled dendritic spine changes on alpha‐motor neurons in ventral horn lamina IX of the spinal cord. This was a postmortem investigation with tissue collected from animals with an established partial‐thickness burn injury that contributed to significant neuropathic mechanical allodynia and heat hyperalgesia (Guo et al., 2018). In this previous study, treatment with romidepsin, a Pak1‐inhibitor, partially attenuated the dendritic spine structural correlates of burn‐induced neuropathic pain. Interestingly, recent work has demonstrated functional relationship between increased nociception and spinal motor reflex excitability. Lee‐Kubli and colleagues have suggested that excessive H‐reflex response may serve as a biomarker for pain (Lee‐Kubli & Calcutt, 2014; Lee‐Kubli, Marshall, Malik, & Calcutt, 2018). Although we have demonstrated a structural relationship between disrupted corticospinal motor tract fibers and reactive sensory afferent plasticity in the dorsal horn (Tan, Chakrabarty, et al., 2012), no report has described the morphological reorganization in synaptic structure in spinal motor neuron pools following an injury‐induced painful condition.

Our primary findings demonstrate an increase in dendritic spine density in ipsilateral spinal motor neurons in burn‐injured animals. Increases in dendritic spines preferentially occurred in dendritic branch regions located closest to the motor neuron soma, a morphological feature indicative of increased neuronal excitability (Bandaru et al., 2015; Tan et al., 2009, 2013). Treatment with romidepsin attenuated these abnormal dendritic spine profiles, returning overall spine density closer to uninjured, Sham levels. Interestingly, in tissue collected from animals following anti‐Pak1 drug withdrawal, dendritic spines remained at near‐normal levels for as long as 4 days (the longest post‐withdrawal interval studied). Taken together, this retrospective study demonstrates for the first time that a second‐degree burn injury can induce potentially adverse motor neuron synaptic plasticity within the spinal cord. When these results are interpreted in combination with our previous functional study (Guo et al., 2018), we conclude that even superficial cutaneous burn injury can create long‐term changes within the spinal cord parenchyma. These structural changes may “lock‐in” the structural substrates necessary for the intractable nature of sensory‐ and motor‐related hyperexcitability disorders—unless treatments target synaptic reorganization at the molecular level.

Our second‐degree burn‐injury model produces an injury profile, wherein epidermal skin damage occurs in the deeper dermis layer, that is, partial‐thickness burn. In our previous studies using a similar burn‐injury model, we reported a reliable development of neuropathic pain that persisted for up to 4 weeks (Chang, Tan, Saab, & Waxman, 2010; Guo et al., 2018; Tan et al., 2013). We also observed an increase in evoked‐ and spontaneous excitability of nociceptive dorsal horn neurons in single‐unit electrical recordings, as well as enlarged unit receptive fields (in cutaneous regions surrounding the burn‐site epicenter) (Chang et al., 2010; Guo et al., 2018; Tan et al., 2013). Taken together, published findings demonstrate that “central sensitization”, that is, increased excitability in spinal circuits, within the burn‐affected spinal cord contributes in part to excessive pain following burn injury (Carlton et al., 2009; Chang & Waxman, 2010; Ji & Woolf, 2001; Kerr et al., 2001).

Importantly, spinal cord hyperexcitability after injury or disease can contribute to both pain and spasticity (Bandaru et al., 2015; Boulenguez et al., 2010; Chang & Waxman, 2010; Ji & Strichartz, 2004; Leon & Dimitrijyevic, 1997; Nielsen, Crone, & Hultborn, 2007; Tan et al., 2009; Waxman & Hains, 2006; Wolpaw, 1994). In the burn‐injury research space, however, it is still not firmly understood how burn trauma can lead to pathological changes in spinal cord excitability. Damaged skin, scar tissue, and injured intracutaneous nerves release inflammatory factors, such as TNF‐alpha, as well as chemokines and trophic factors. As part of the healing process, many of these molecular factors can remotely signal long‐distances and alter neuronal excitability (Hansen et al., 2013, 2016; Zhao, Waxman, & Hains, 2007). Injured peripheral nerve afferents have been shown to misexpress sodium channels following burn‐ or mechanical nerve injury. Abnormal sodium channel expression can significantly alter nociceptive excitability associated with painful conditions (Bennett, Clark, Huang, Waxman, & Dib‐Hajj, 2019; Eaton, 2003; Ji & Strichartz, 2004; Samad et al., 2013; Shields et al., 2012; Wang et al., 2016). In the spinal reflex pathway, injured primary afferents may also potentiate changes within the central motor system (Bandaru et al., 2015; D'Amico, Condliffe, Martins, Bennett, & Gorassini, 2014; Sjolund, 2002). Interestingly, emerging evidence has shown that sodium channel misexpression may also occur within the spinal cord ventral horn motor pools (Brocard et al., 2016; Li & Bennett, 2003; Li, Gorassini, & Bennett, 2004). For example, increased mRNA expression of tetrodotoxin (TTX)‐sensitive sodium channels, such as Nav1.3 and Nav1.7, in ipsilateral spinal motor neuron pools has been reported following axotomy (Fukuoka, Kobayashi, & Noguchi, 2010). Finally, astrocyte processes are intimately associated with synaptic dendritic spines, and have a direct functional effect on changing the excitability of neurons and the quality of synaptic transmission (Bourne & Harris, 2008; Brockett, LaMarca, & Gould, 2015; Nishida & Okabe, 2007; Perez‐Alvarez, Navarrete, Covelo, Martin, & Araque, 2014). Because our current retrospective study does not directly monitor changes in motor neuron physiology or behavioral outcome, we can only speculate that our observations of dendritic spine remodeling are one of the many elements involved in severe burn‐injury pathology. In general, burn injury is a multifaceted insult which leads to long‐lasting changes in neuronal and nonneuronal tissues.

Our study raises the important question of the role of dendritic spine remodeling on motor neurons following burn injury. The majority of excitatory synapses in the spinal cord occur on dendritic spines (Calabrese et al., 2006; McKinney, 2010; Niesmann et al., 2011). Dendritic spines are microscopic‐sized, postsynaptic structures that contribute to modifying synaptic transmission within circuits. Although there is a notable lack of preclinical study in the burn‐injury field, especially with regard to chronic neurological complications, the available literature shows a significant adverse effect of burn injury on spinal motor neuron structure and function. In a clinical case study, electrical injury and superficial burn trauma to the forearm in a human patient were reported to lead to progressively increased spastic symptoms in the lower extremities, that is, hyperreflexia, that was then followed by progressive motor neuron death, exhibiting ALS‐like neuropathology, and multi‐limb paralysis over the course of several months (Sirdofsky et al., 1991). Indeed, motor neurons with increased dendritic spine density demonstrated improved cell survival in a mouse model of ALS; suggesting that dendritic spine proliferation is predictive of a neuroprotective response (Fogarty et al., 2016).

Alternatively, as shown in several animal models, activity loss, for example, monocular deprivation or injury, contributes to a “destabilization” of synapses, including dendritic spine structure (Berry & Nedivi, 2017; Kim et al., 2006; Villa et al., 2016; Zuo, Yang, Kwon, & Gan, 2005). For inhibitory synapses, monocular deprivation leads to a transition to a new steady‐state, whereby inhibitory synapses are less likely to be present (Villa et al., 2016). For dendritic spines, which are primarily excitatory, a greater number of thin‐elongated dendritic spines appear on cortical pyramidal motor neurons following spinal cord injury. Notably, thin‐elongated dendritic spines are more dynamic, appearing and disappearing at a greater rate than more mature, mushroom‐shaped spines. Thus, injury leads to changes in dendritic spine structure indicative of a shift toward a higher dynamic steady‐state—for example, greater plasticity follows injury (Kim et al., 2006). In the same injury model, restoring trophic support and environmental enrichment—which can stimulate intrinsic sensory *and* motor activity throughout the CNS—restored the pre‐injury pattern of dendritic spine morphologies in the motor cortex (Berrocal et al., 2007; Dunlop, 2008; Florence et al., 2001; Kim, Dai, McAtee, & Bregman, 2008). Taken together, in our model of burn injury, dendritic spine remodeling may therefore also be a consequence of the loss of activity and a transition to a new dynamic state in spinal motor neurons.

In our published studies, we have identified a common structural motif of dendritic spine morphology strongly associated with post‐injury hyperexcitability disorders, for example, spasticity (Bandaru et al., 2015; Guo et al., 2018; Tan et al., 2013; Tan & Waxman, 2015; Zhao et al., 2016). As shown in our current retrospective study, we observed two features of abnormal dendritic spine remodeling in ipsilateral ventral horn motor neurons: I) increased spine density, particularly mushroom‐shaped dendritic spines, and II) a greater distribution of spines in regions closer to the cell body. These changes are similar to those we observed in our previous report in sensory neurons (Guo et al., 2018). Although motor neurons have an overall lower density of dendritic spines compared with sensory neurons, following burn injury, both neuronal types exhibit a significant increase in spine density (see Table 2). In light of other published studies, these changes could have physiological implications for sensory or motor neuron circuits. As shown in several models and in silico, increased dendritic spine density and redistribution could contribute to pathophysiological changes in circuit excitability associated with a number of neurological disorders (Halpain, Spencer, & Graber, 2005; Tan et al., 2009, 2013; Zhao et al., 2016). In our previous burn‐injury studies, we show both an increase in cutaneous evoked single‐unit nociceptive excitability in the spinal cord dorsal horn and lower pain threshold in behavioral assays (Chang et al., 2010; Guo et al., 2018; Tan et al., 2013). This retrospective study of motor neurons did not specifically measure motor evoked activity; however, we have shown no significant difference in gross motor strength or rearing behavior as compared with animal without injury (Guo et al., 2017). Future next steps would be to determine whether dendritic spine remodeling on spinal cord motor neurons following burn injury has lasting physiological implications that manifest at the behavioral level.

Here, we further investigated whether disruption of Pak1 signaling would affect motor neuron dendritic spine morphology following burn injury. We have shown that Pak1‐inhibition with romidepsin treatment could significantly correct abnormal dendritic spines on nociceptive dorsal horn neurons, and reduce the presence of neuropathic pain after burn injury (Guo et al., 2018). Pak1 is localized at dendritic spines, interacts with postsynaptic density proteins, PSD‐95 and f‐actin, and may directly mediate spine maturation and stabilization (Hayashi, Ohshima, Hashimoto, & Mikoshiba, 2007). Pak1 mutation and dysregulation contributes malformed dendritic spines observed in a broad spectrum of neuropsychiatric diseases (Kichina et al., 2010; Ma et al., 2012; Nikolic, 2008). We have shown significant reduction in dendritic spine densities on dorsal horn sensory neurons (Table 2; Guo et al., 2017). Although it is not yet known how burn injury triggers dendritic spine remodeling, the present study shows that romidepsin treatment has some durable efficacy in blocking the development of abnormal spine morphologies in spinal motor neurons after burn injury.

There are two main caveats to our study. First, we did not perform any motor functional studies in this anatomical study. We can therefore only speculate on functional implications of dendritic spine remodeling on motor neurons after burn injury. Second, although we observed no effect of systemic romidepsin treatment on inflammation in any region of the spinal cord (see previous study; (Guo et al., 2018)), we cannot rule out the possibility of an effect of the drug in the PNS. Peripheral afferent plasticity following injury or disease may influence spinal motor neuron structure and function (Bandaru et al., 2015; Lee‐Kubli & Calcutt, 2014; Tan, Chakrabarty, et al., 2012). It is possible that Pak1 inhibition had a mode‐of‐action on extra‐spinal elements, which indirectly affected our dendritic spine observations in spinal motor neurons. Nonetheless, it is clear that romidepsin treatment has a predictable corrective effect upon a morphological correlate of dysfunction in the spinal cord.

## 5 CONCLUSION

A major challenge in burn injury research is lack of clarity in the underlying mechanisms associated with secondary neurological complications. Here, we show for the first time that Pak1‐mediated dendritic spine dysgenesis occurs on spinal alpha‐motor neurons following a partial‐thickness burn injury. Our findings underscore the need for additional research into understanding the cellular‐molecular relationship between dendritic spine remodeling and chronic complications of burn trauma.

## CONFLICT OF INTEREST

The authors declare no conflict of interest.
